# Utilizing ChatGPT for Curriculum Learning in Developing a Clinical Grade Pneumothorax Detection Model: A Multisite Validation Study

**DOI:** 10.3390/jcm13144042

**Published:** 2024-07-10

**Authors:** Joseph Chang, Kuan-Jung Lee, Ti-Hao Wang, Chung-Ming Chen

**Affiliations:** 1 Department of Biomedical Engineering, College of Medicine and College of Engineering, National Taiwan University, No. 1, Sec. 1, Jen-Ai Road, Taipei 100, Taiwan; 2 EverFortune.AI Co., Ltd., Taichung 403, Taiwan; 3 Department of Medicine, China Medical University, Taichung 404, Taiwan; 4 Department of Radiation Oncology, China Medical University Hospital, Taichung 404, Taiwan

**Keywords:** artificial intelligence, deep learning, curriculum learning, pneumothorax

## Abstract

**Background**: Pneumothorax detection is often challenging, particularly when radiographic features are subtle. This study introduces a deep learning model that integrates curriculum learning and ChatGPT to enhance the detection of pneumothorax in chest X-rays. **Methods**: The model training began with large, easily detectable pneumothoraces, gradually incorporating smaller, more complex cases to prevent performance plateauing. The training dataset comprised 6445 anonymized radiographs, validated across multiple sites, and further tested for generalizability in diverse clinical subgroups. Performance metrics were analyzed using descriptive statistics. **Results**: The model achieved a sensitivity of 0.97 and a specificity of 0.97, with an area under the curve (AUC) of 0.98, demonstrating a performance comparable to that of many FDA-approved devices. **Conclusions:** This study suggests that a structured approach to training deep learning models, through curriculum learning and enhanced data extraction via natural language processing, can facilitate and improve the training of AI models for pneumothorax detection.

## 1. Introduction

Pneumothorax is a thoracic disease defined as the presence of air in the pleural space between the lungs and the chest wall. This condition has a significant incidence in both primary spontaneous pneumothorax and secondary spontaneous pneumothorax; its detection is critical, especially given that the high risk and recurrence in the secondary form is associated with underlying lung diseases, complicating both diagnosis and management [1,2]. Secondary spontaneous pneumothorax, in particular, is a potentially life-threatening condition that demands immediate clinical attention, as recommended by the American College of Chest Physicians (ACCP) [3,4,5]. Chest X-ray (CXR) imaging remains the primary diagnostic tool for identifying pneumothorax, offering quick assessment. However, the interpretation of these images can be challenging; subtle signs of pneumothorax may be missed, with a reported miss rate of up to 20% for occult pneumothoraces [6]. Variabilities in thoracic disease presentations and patient postures can distort CXR images, complicating the detection process further [7,8,9,10].

In recent years, advances in deep learning (DL) have shown promising improvements in the automated detection of pneumothorax or other thoracic conditions, with studies leveraging large, open-source datasets like ChestX-ray14 (NIH) and CheXpert that demonstrate promising performances [11,12,13]. However, the effectiveness of these advances can be compromised by a failure to differentiate between simple and hard samples during training, a distinction crucial for achieving robust, generalizable models. This issue is compounded by the common practice of internal validation, which may not adequately represent real-world clinical settings and could lead to potential overfitting and limited generalizability [14,15,16].

To address these challenges, this study proposes a novel integration of ChatGPT with a curriculum learning framework to refine the training of DL model for pneumothorax detection. Recent studies have demonstrated that ChatGPT can effectively interpret and handle clinical data to support medical education and clinical practice, demonstrating its ability in managing complex medical terminologies [17,18,19,20,21]. Utilizing ChatGPT’s capabilities, we extracted and curated “hard samples” from radiology reports. These hard samples were based on pneumothorax size, a critical factor according to the ACCP guidelines for assessing severity and treatment pathways [22,23]. This curated approach enhances the training samples based on difficulty and informativeness, which is aligned with clinical realities where smaller pneumothoraces, although harder to detect, carry significant clinical risks if missed [24].

The utilization of curriculum learning (CL), a proven educational methodology, in our model’s training regimen allows for a systematic increase in diagnostic challenges, starting with larger, easily detectable pneumothoraces and gradually incorporating smaller, more subtle cases [25,26,27]. This progression mirrors the clinical urgency to detect all pneumothoraces, irrespective of size, and promotes a more natural, human-like learning process in training the AI algorithm. Furthermore, the integration of ChatGPT in extracting pneumothorax size from unstructured radiology text addresses the potential to enrich datasets for DL training, providing a novel approach to creating more robust and clinically relevant models.

This study aimed to develop an AI algorithm for pneumothorax detection by leveraging the data extraction capabilities of ChatGPT and employing strategic curriculum learning. The results were then validated on external multisite and multireader data to ensure the performance of the DL model for potential use in clinical practice.

## 2. Materials and Methods

### 2.1. Patient Selection and Classification

**Figure 1:**
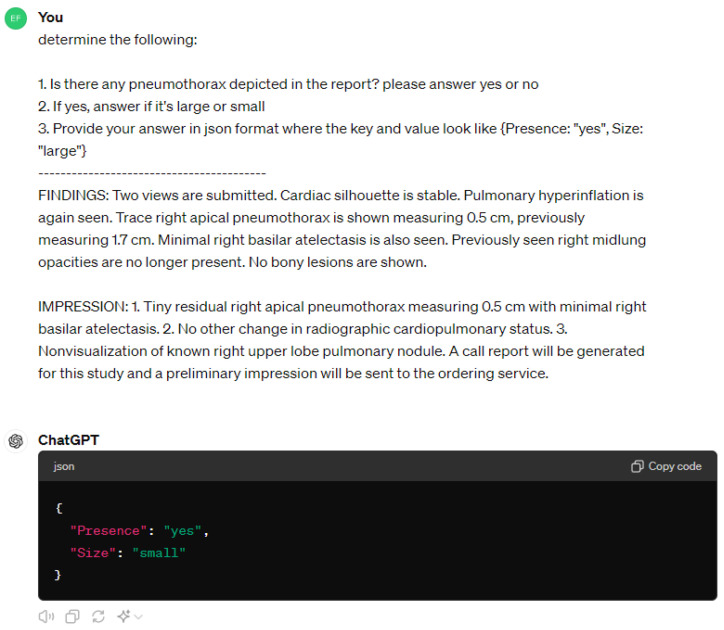
Example of obtaining pneumothorax information with ChatGPT.

This retrospective study used data that were fully de-identified, anonymized and accessed under IRB CMUH113-REC2-049 with waived consent. For the development of the AI algorithm, a dataset comprising 6445 anonymized chest radiographs was utilized. These images were consecutively collected over a span of time from 2006 to 2018. Accompanying these radiographs, corresponding radiology reports were processed to extract clinically relevant information utilizing the advanced natural language processing capabilities of ChatGPT4, which was created by OpenAI. The primary task assigned to ChatGPT was to determine the presence of pneumothorax from the text of the radiology reports. Additionally, for cases where pneumothorax was identified, ChatGPT was further tasked with classifying the condition as either “large” or “small” based on descriptors found within the reports, as shown in Figure 1 below. This classification acts as a preliminary result to help differentiate simple and hard samples in an unsupervised manner to help structure the subsequent training phases of the AI model, aligning with the curriculum learning framework that progresses from more straightforward to more challenging cases.

### 2.2. Deep Learning Model Building

In this study, we utilized EfficientNetB3 as the backbone architecture for our deep learning model. EfficientNetB3 offers a balanced structure that enhances performance without significantly increasing computational demand, making it suitable for medical image analysis [28,29]. Its architecture, which scales uniformly with depth, width, and resolution, is ideal for detailed image features, such as those required for detecting varying sizes of pneumothorax [30,31]. The EfficientNetB3 model was optimized using the Adam optimizer, with an initial learning rate of 0.001, which was reduced by a factor of 10 whenever the validation loss plateaued for over 10 epochs. The model was trained using a binary cross-entropy loss function. To reduce overfitting and ensure generalization across diverse radiographic data, we implemented dropout at a rate of 0.5 and L2 regularization. Data augmentation techniques, including random rotations (up to 30 degrees) and horizontal flips, were applied to increase the diversity of training images. Training was conducted over 400 epochs with a batch size of 32, utilizing early stopping based on validation loss to prevent overtraining.

The training process was structured around a CL framework designed to adaptively introduce complexity, where the presence of pneumothorax and sizes were based solely on the results from ChatGPT. Initially, the model was trained exclusively on datasets labeled as “large” pneumothorax for cases where the condition was present; these are generally easier to identify due to their distinct visual characteristics. This initial phase was set up such that positive cases in each batch were composed of larger pneumothorax cases to establish a baseline proficiency in recognizing clearer and more distinguishable patterns. As the model’s performance on these cases improved, we gradually introduced small pneumothoraces into the batches. The introduction was controlled and incremental, starting with a 5% inclusion of small pneumothorax cases; this percentage was increased by 5% each time the model achieved a predetermined performance threshold on the validation set. The progression from large to mixed batches was governed by an adaptive feedback mechanism, wherein the model’s performance on periodically evaluated validation sets determined the timing and rate of transition. To align with the US Food and Drug Administration (FDA) recommendations regarding approved device performances in developing a clinical-grade model, the transition criteria were designed such that the model was required to achieve and maintain an area under the receiver’s operating characteristic curve (AUC) greater than 95%, as well as both sensitivity and specificity greater than 80%, before each transition of introducing additional small pneumothorax cases [32].

**Figure 2:**
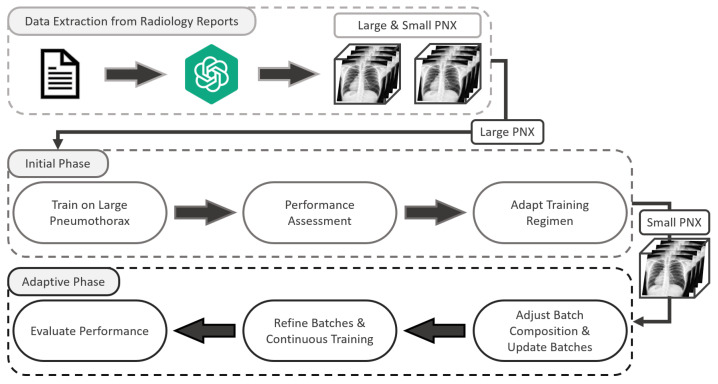
The curriculum training framework for pneumothorax (PNX) detection.

This curriculum-based approach is inspired by previous studies, where incremental learning has shown to improve the efficiency of model training processes by initially focusing on less complex examples [33,34,35]. In our implementation, this principle was applied by using a dynamic adjustment strategy, where the ratio of large to small pneumothorax cases in training batches was continually adapted based on the model’s current learning state [36,37,38]. This approach ensures that the model is not overwhelmed by the complexity of smaller, subtler pneumothorax cases, thereby enhancing its ability to develop a comprehensive analytic capability over time. The overall CL framework design is shown in Figure 2.

### 2.3. External Multisite Validation Data Collection

**Figure 3:**
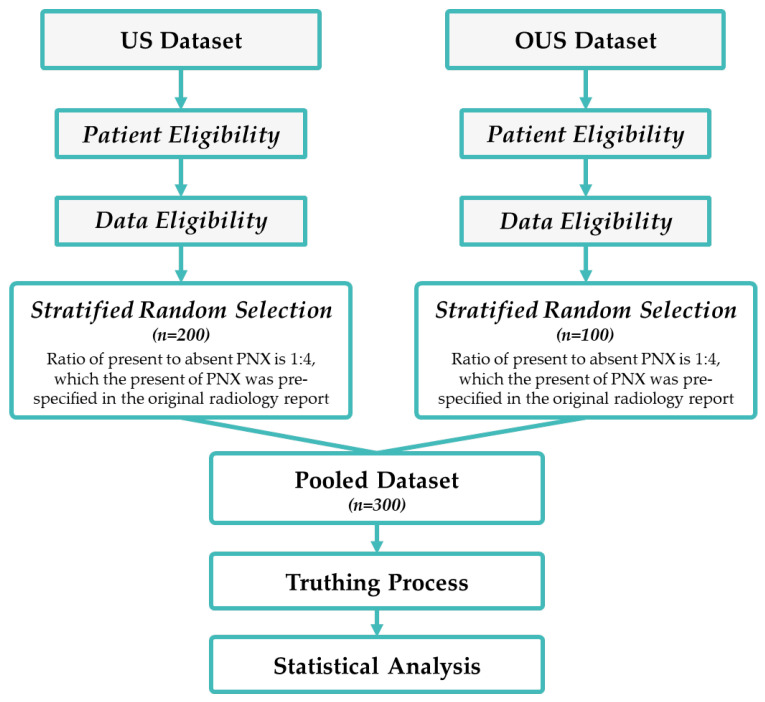
Flowchart of the validation workflow.

A total of 300 anonymized chest radiographs were consecutively collected between 2016 and 2021 from 3 clinical sites in the U.S. and from 1 clinical site in Taiwan (OUS). The dataset was generated from 9 different manufacturers of radiologic data sources, including Samsung Electronics, Shimadzu, Toshiba, GE Healthcare, Swissray, Philips Medical System, Konica Minolta, Canon Inc., Fujifilm Corporation, etc. The radiographs were collected following the inclusion and exclusion criteria, with stratification depending on whether the patient received thoracentesis, as shown in Figure 3.

In this study, all patients were required to be at least 18 years old, applicable to both males and females. For chest X-ray images, the inclusion criteria emphasized clarity and diagnostic value, requiring images to feature an unobscured chin, arms not covering the chest’s lateral walls, symmetrically placed sternoclavicular joints relative to the spinal column, horizontal clavicles with no more than ten ribs visible above the diaphragm, and a diaphragm intersecting between the 5th and 7th anterior ribs. Additionally, clear visibility of ribs and the thoracic cage over the heart area, along with distinctly visible vascular lung markings, were mandatory. Any images that met the following criteria were excluded: obscured chins, arms obscuring the chest’s lateral walls, asymmetrically placed sternoclavicular joints, non-horizontal clavicles, more than ten visible ribs above the diaphragm, inappropriate diaphragm intersection, or compromised visibility of ribs and thoracic cage. These criteria ensured that the X-rays used were of high quality and suitable for accurate analysis.

### 2.4. Ground Truth Definition

The ground truthing of this assessment study included three U.S. board-certified ex-pert radiologists reviewing the radiographs and assigning whether a pneumothorax was present in each image. These radiologists all had the U.S. American Board of Radiology (ABR) board certification in Diagnostic Radiology, with more than 10 years of experience in assessing chest X-rays and conducting a high volume (greater than 75 cases per week) of CXR assessments. For each case, each radiologist was asked to provide the following information: whether pneumothorax is present or absent; the size (large ≥ 3 cm and small < 3 cm), which was measured from the thoracic cupola to the lung apex and defined by the ACCP guidelines; the location, including right upper lobe (RUL), right middle lobe (RML), right lower lobe (RLL), left upper lobe (LUL), and left lower lobe (LLL); and any additional comments the radiologist would like to provide about the case. The presence, size, and location of pneumothorax were determined based on the majority agreement of the three U.S. board-certified expert radiologists who reviewed the radiographs independently, and this information was further defined as the ground truth (GT).

### 2.5. Statistical Analysis

Our study utilized Python 3.6 and R 4.0.2 for data processing and statistical analyses. The chi-square test assessed the independence of categorical variables, examining whether significant associations existed between them. To validate the AI algorithm’s sensitivity, specificity, and AUC, One-Sample Z tests were employed, comparing the algorithm’s results against the GT. We calculated 95% confidence intervals (95% C.I.) for sensitivity, specificity, and AUC to comprehensively evaluate the algorithm’s ability to detect pneumothorax. DeLong’s method was applied to compute the 95% CIs for the AUC, a technique specifically suited for correlated ROC curves. This method corrects for the correlation between AUC estimates, ensuring a rigorous assessment of diagnostic accuracy. For sensitivity and specificity, Wilson’s score method was used to calculate 95% CIs, offering precise intervals for binomial proportions, which is especially valuable in scenarios with small sample sizes or extreme event rates. This study used an upper-tail test with a significance level (Type I error, α) of 2.5% to test our hypotheses. This framework supported the AI algorithm’s generalizability across various subpopulations, including different genders (male, female), age groups (18–34, 35–49, 50–64, above 65 years), and data sources (U.S., Taiwan). We also examined performance variations among different manufacturers (Samsung Electronics (Suwon-si, Republic of Korea), Shimadzu (Kyoto, Japan), Toshiba (Tokyo, Japan), etc.), and analyzed sensitivity based on pneumothorax size (small, large) and anatomical location (RUL, RML, RLL, LUL, LLL). Potential confounders, such as image quality issues and unrelated radiologic findings, were also considered to determine their impact on the AI’s performance in detecting pneumothorax.

## 3. Results

### 3.1. Patient Characteristics

During the training process, ChatGPT processed each radiograph’s corresponding radiology report, and out of the 6445 chest radiographs, a total of 2554 radiographs were identified to contain pneumothoraces. Among these cases, ChatGPT classified 773 as “small” pneumothoraces and 1781 as “large” pneumothoraces. This classification enabled the structuring of the training phases of the AI model, aligning with the curriculum learning framework to ensure that the model was exposed to varying complexities in a more meaningful order.

To validate the model’s generalizability, external multisite data of a total of 300 chest X-ray images that met the inclusion criteria were consecutively collected for the study. Among them, 162 (54.0%) were male and 138 (46.0%) were female. The mean (standard deviation, SD) age was 49.5 (16.3) years. The case distribution was as detailed in Table 1 based on the presence of pneumothorax or not.

### 3.2. Evaluation of AI Performance

The performance metrics used were sensitivity, specificity, and the AUC. In order to assess whether the model performance has potential for future clinical use, we set the performance goals to match closely with the ones used in many US FDA-approved devices, where the sensitivity/specificity should reach over 0.8 and the AUC should be greater or equal to 0.95 [32]. As shown in Table 2, the One-Sample Z tests showed that the sensitivity and specificity both exceeded 0.8; the AUC also exceeded 0.95. The sensitivity of the AI algorithm was 0.97, with a 95% CI of [0.90, 0.99]; the specificity was 0.97, with a 95% CI of [0.94, 0.99]; and the AUC was 0.98, with a 95% DeLong’s CI of [0.96, 1.00]. Overall, the agreement between the AI algorithm and the GT met the performance goal of exceeding 0.8 in both sensitivity and specificity and of exceeding 0.95 in AUC, compared with the GT.

**Figure 4:**
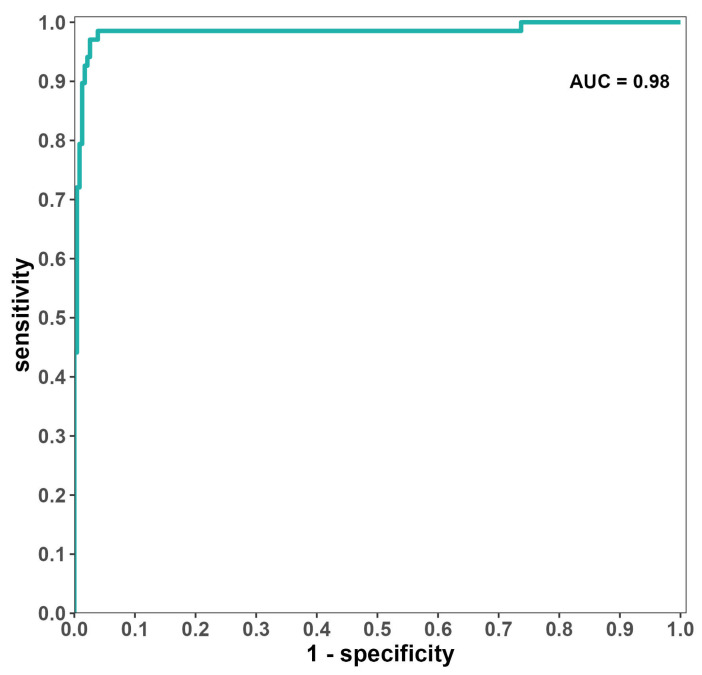
ROC curve of the AI algorithm.

The ROC curve (Figure 4) offers a graphical representation of the AI algorithm’s performance, illustrating how the AUC encompasses the entire two-dimensional space under the ROC curve at all classification thresholds, spanning from (0, 0) to (1, 1), with a value of 0.98. This high AUC value for the 300 evaluated cases underscores the algorithm’s strong classification capability.

### 3.3. Subgroup Analysis Results

We conducted subgroup analyses to evaluate the AI algorithm’s performance across various subpopulations (Table 3 and Table 4). This examination against the GT for specific groups ensures the AI system’s reliability and effectiveness across a wide range of patient demographics.

This study’s results show that all subpopulations achieved a high performance in detecting pneumothorax, with sensitivity ranging from 0.93 to 1.00 and specificity between 0.91 and 1.00. These results highlight the AI algorithm’s robustness in evaluating pneumothorax across various patient demographics and clinical conditions, effectively identifying different sizes of pneumothorax.

### 3.4. Ablation Study Results

**Figure 5:**
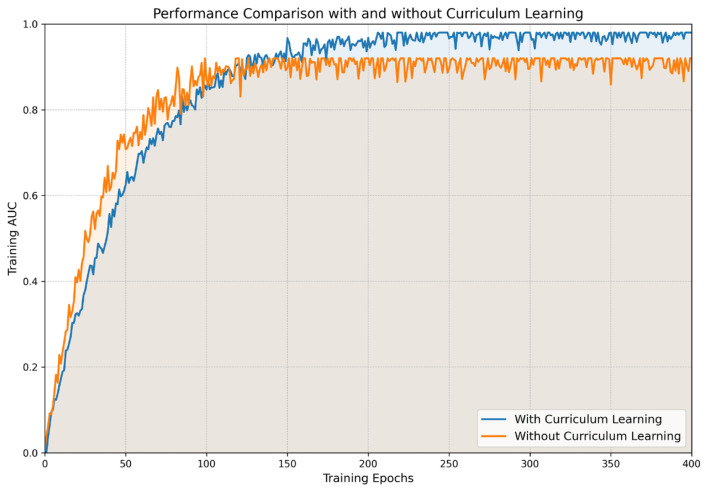
Performance comparison with and without curriculum learning.

The CL framework was designed to incrementally introduce images in a more gradual order, in this case, by starting with easier samples such as larger pneumothorax and adaptively moving into more complex subtle cases. The external multisite validation results, as shown in Figure 5, demonstrate that the CL framework achieved a superior performance compared to the traditional training method. Specifically, CL achieved a higher overall AUC of 0.98, whereas the traditional method, without the graduated complexity of curriculum learning, reached an AUC of 0.92 on the external validation data. Initially, models trained with only large pneumothorax exhibited a slower improvement in performance in the early epochs as the model began with simpler, more obvious examples, allowing for the consolidation of foundational knowledge before progressively introducing more complex examples. In contrast, the traditional training method exposed the model to the full diversity of the dataset from the beginning, including both large and small pneumothorax cases, resulting in faster initial improvements. However, after the initial phase, the model with curriculum learning showed a sharper increase in performance as more complex data were introduced. This is indicative of the model’s enhanced tuning to foundational patterns, enabling it to generalize more effectively from increasingly difficult data. Conversely, the traditional method, per curve, tended to plateau earlier as the model potentially began to overfit or exhaust the learning from the diverse training set.

### 3.5. Comparison of CADt Algorithms

As shown in Table 5, we conducted a comparative analysis of our algorithm versus existing US FDA-approved devices, specifically designed for pneumothorax detection. Key performance indicators included sensitivity, specificity, and the AUC. Our primary objectives were to achieve a minimum sensitivity and specificity of 0.8 and an AUC of 0.95. The results of our proposed algorithm achieved a sensitivity of 0.97, a specificity of 0.97, and an AUC of 0.98, thereby meeting and exceeding the benchmarks set by leading FDA-approved solutions for pneumothorax detection [32].

## 4. Discussion

This study demonstrates the utilization of ChatGPT with a curriculum learning framework, enhancing the potential of the development of a more robust pneumothorax detection model for potential clinical use. Our multisite validation demonstrated that leveraging advanced natural language processing tools could help build a more clinically tailored CL framework to handle varied complexities, such as different sizes of pneumothoraces. The results reveal that the CL framework can outperform traditional training methods. Systematically introducing more subtle and clinically challenging cases into the training process allowed the model not only to achieve higher performance but also to ensure robustness and generalizability across diverse subgroups. This improvement is particularly notable in the detection of small pneumothoraces, which are often more challenging to diagnose due to their subtle radiographic features.

The incremental introduction of complexity in the training set proved crucial in enhancing the model’s diagnostic capabilities. Starting with simpler, large pneumothorax cases allowed the model to establish a stronger foundation model. This strategic approach was followed by the introduction of smaller, more complex pneumothorax cases, which challenged the model’s learning depth and adaptability. Such a structured approach prevented the early plateauing of model performance, a common issue in traditional training methods where the model is exposed to a heterogeneous mix of cases from the beginning. The model’s performance in detecting both large and small pneumothoraces on external multisite and multireader ground truth results suggests its potential for real-world clinical application. In clinical practice, the ability to accurately identify small pneumothoraces is crucial, as these cases are more likely to be overlooked yet carry significant risks if undetected.

Previous studies have also shown promising results by integrating LLMs such as ChatGPT to help streamline the analysis of clinical reports. For example, Doshi et al. [39] demonstrated that ChatGPT and similar LLMs are adept at simplifying radiology reports, which can help enhance patient understanding. Similarly, a study by Tan et al. [40] demonstrated the effectiveness of LLMs in inferring cancer disease responses from radiology reports. The results of this study further confirm the potential of LLMs to process and analyze large clinical datasets with minimal human intervention and extend the use case and scope of curriculum learning to include more varied and complex cases.

This study has certain limitations and potential areas of future work. One area of enhancement would be the integration of a multimodal deep learning approach. Our study primarily leveraged text-based radiology reports; however, incorporating radiology images alongside reports could further enhance the model’s performance by capturing more comprehensive and nuanced features. Another consideration is the diversity of the sample complexity. While this study considered pneumothorax size as a primary factor in defining sample complexity, it is also worth exploring other features beyond size that may complicate diagnosis. Features such as atypical presentations, overlapping pathologies, or subtle radiographic signs could be extracted from radiology reports to provide a more holistic training. Additionally, incorporating difficult negative cases, such as conditions that mimic pneumothorax into the training process, could also enhance the model’s discriminative capabilities, reducing false positives. In terms of limitations, the model’s performance was dependent on the quality and comprehensiveness of the data extracted by ChatGPT, indicating that the model’s effectiveness was as good as the data it was trained on. Future studies could explore the integration of additional data sources and perhaps a more extensive range of text-based clinical documentation to further enhance the model’s training. Additionally, ongoing refinements in the CL algorithm could provide more dynamic adjustments to training complexities based on real-time performance metrics.

Furthermore, the integration of curriculum learning with the capabilities of ChatGPT in extracting and curating clinical information offers a novel approach in training models with data that are not only more insightful but also more clinically relevant. This model’s performance in the multisite study suggests that such an approach could be replicated and scaled to other tasks within the field.

## 5. Conclusions

In conclusion, in this study, we developed a novel approach to integrating ChatGPT with a CL framework by creating a pneumothorax detection model. By methodically enhancing the training process with the inclusion of clinical information extracted from radiology reports, the model was able to achieve an overall AUC of 0.98, thus surpassing traditional training methods. The multisite external validation confirms the model’s effectiveness across different subgroups, demonstrating its robustness and its potential for future clinical use. Lastly, the findings suggest that a robust AI model such as this one could potentially enhance clinical practice by improving the detection of pneumothorax, particularly smaller instances, to reduce the risk associated with delayed or missed diagnosis.

## Figures and Tables

**Table 1:** Basic characteristics for 300 external validation datasets.

	Pneumothorax Absent (*n* = 232)	Pneumothorax Present (*n* = 68)	*p*-Value
Gender			0.0607
Female	114	24	
Male	118	44	
Age			<0.0001
18–34 y/o	37	25	
35–49 y/o	68	13	
50–64 y/o	99	15	
above 65 y/o	28	15	
Data Source			0.4072
U.S.	158	42	
Taiwan	74	26	
X-ray Manufacturer			<0.0001
Samsung Electronics	137	10	
Shimadzu	30	15	
Toshiba	44	11	
Others *	21	32	

* Other manufacturers include GE Healthcare (*n* = 16), Swissray (*n* = 14), Philips Medical System (*n* = 9), Konica Minolta (*n* = 9), Canon Inc. (*n* = 3), and Fujifilm Corporation (*n* = 2).

**Table 2:** Performance of the AI algorithm.

Metrics	Performance	95% C.I.
Sensitivity	0.97	(0.90, 0.99)
Specificity	0.97	(0.94, 0.99)
AUC	0.98	(0.96, 1.00)

**Table 3:** Performance of the AI algorithm in each subpopulation.

Characteristics	Sensitivity (Wilson’s CI)	Specificity (Wilson’s CI)	AUC (DeLong’s CI)
Gender			
Female	1.00 (0.86, 1.00)	0.97 (0.93, 0.99)	0.99 (0.98, 1.00)
Male	0.95 (0.85, 0.99)	0.97 (0.92, 0.99)	0.98 (0.95, 1.00)
Age			
18–34 y/o	0.96 (0.80, 1.00)	0.97 (0.86, 1.00)	0.97 (0.92, 1.00)
35–49 y/o	1.00 (0.77, 1.00)	0.97 (0.90, 0.99)	1.00 (0.99, 1.00)
50–64 y/o	0.93 (0.70, 1.00)	0.97 (0.91, 0.99)	0.99 (0.98, 1.00)
above 65 y/o	1.00 (0.80, 1.00)	0.96 (0.82, 1.00)	0.98 (0.93, 1.00)
Data Source			
U.S.	0.95 (0.84, 0.99)	0.96 (0.92, 0.98)	0.97 (0.93, 1.00)
Taiwan	1.00 (0.87, 1.00)	0.99 (0.93, 1.00)	1.00 (0.99, 1.00)
Manufacturer			
Samsung Electronics	1.00 (0.72, 1.00)	0.99 (0.95, 1.00)	1.00 (1.00, 1.00)
Shimadzu	1.00 (0.80, 1.00)	1.00 (0.89, 1.00)	1.00 (1.00, 1.00)
Toshiba	1.00 (0.74, 1.00)	0.98 (0.88, 1.00)	1.00 (0.99, 1.00)
Others *	0.94 (0.80, 0.98)	0.91 (0.80, 0.92)	0.90 (0.81, 1.00)

* Other manufacturers including GE Healthcare, Swissray, Philips Medical System, Konica Minolta, Canon Inc and Fujifilm Corporation.

**Table 4:** Sensitivity of the AI algorithm for each pneumothorax size and location.

Characteristics	Sensitivity (Wilson’s CI)
Pneumothorax Size	
Small	0.97 (0.87, 1.00)
Large	0.97 (0.83, 1.00)
Pneumothorax Location	
RUL	1.00 (0.90, 1.00)
RML	1.00 (0.78, 1.00)
RLL	1.00 (0.83, 1.00)
LUL	0.97 (0.83, 1.00)
LLL	0.96 (0.84, 1.00)

**Table 5:** Performance comparison against FDA 510(k)-approved CAD(t) AI algorithms [32].

Devices	Sensitivity	Specificity	AUROC
HealthPNX	0.93	0.92	0.98
Critical Care Suite	0.84	0.93	0.96
Red Dot	0.94	0.87	0.97
AIMI-Triage CXR	0.92	0.90	0.96
Ours	0.97	0.97	0.98

## Data Availability

The datasets analyzed in the current study are not publicly available due to ethical restrictions and the proprietary nature of the study.
